# B-Cell Dysregulation in Crohn's Disease Is Partially Restored with Infliximab Therapy

**DOI:** 10.1371/journal.pone.0160103

**Published:** 2016-07-28

**Authors:** Wilhelmina M. C. Timmermans, Jan A. M. van Laar, Tim B. van der Houwen, Lieke S. J. Kamphuis, Sophinus J. W. Bartol, King H. Lam, Rob J. Ouwendijk, Miles P. Sparrow, Peter R. Gibson, P. Martin van Hagen, Menno C. van Zelm

**Affiliations:** 1 Department of Internal Medicine, Erasmus MC, Rotterdam, The Netherlands; 2 Department of Immunology, Erasmus MC, Rotterdam, The Netherlands; 3 Department of Pathology, Erasmus MC, Rotterdam, The Netherlands; 4 Department of Gastroenterology, Ikazia Hospital, Rotterdam, The Netherlands; 5 Department of Gastroenterology, Alfred Hospital, Monash University and Alfred Health, Melbourne, VIC, Australia; 6 Department of Immunology and Pathology, Monash University, Melbourne, VIC, Australia; COCHIN INSTITUTE, Institut National de la Santé et de la Recherche Médicale, FRANCE

## Abstract

**Background:**

B-cell depletion can improve a variety of chronic inflammatory diseases, but does not appear beneficial for patients with Crohn’s disease.

**Objective:**

To elucidate the involvement of B cells in Crohn’s disease, we here performed an ‘in depth’ analysis of intestinal and blood B-cells in this chronic inflammatory disease.

**Methods:**

Patients with Crohn’s disease were recruited to study B-cell infiltrates in intestinal biopsies (n = 5), serum immunoglobulin levels and the phenotype and molecular characteristics of blood B-cell subsets (n = 21). The effects of infliximab treatment were studied in 9 patients.

**Results:**

Granulomatous tissue showed infiltrates of B lymphocytes rather than Ig-secreting plasma cells. Circulating transitional B cells and CD21^low^ B cells were elevated. IgM memory B cells were reduced and natural effector cells showed decreased replication histories and somatic hypermutation (SHM) levels. In contrast, IgG and IgA memory B cells were normally present and their Ig gene transcripts carried increased SHM levels. The numbers of transitional and natural effector cells were normal in patients who responded clinically well to infliximab.

**Conclusions:**

B cells in patients with Crohn’s disease showed signs of chronic stimulation with localization to granulomatous tissue and increased molecular maturation of IgA and IgG. Therapy with TNFα-blockers restored the defect in IgM memory B-cell generation and normalized transitional B-cell levels, making these subsets candidate markers for treatment monitoring. Together, these results suggest a chronic, aberrant B-cell response in patients with Crohn’s disease, which could be targeted with new therapeutics that specifically regulate B-cell function.

## Introduction

The human intestinal tract contains a complex interplay between commensal bacteria, food antigens and the host immune system to limit inflammation, while preventing the translocation of intestinal microbiota. This delicate balance is disrupted in Crohn’s disease, a chronic inflammatory disease characterized by transmural inflammation of the gastrointestinal tract [1]. The pathogenesis of Crohn’s disease is of complex nature with genetic susceptibility and dysfunction of mucosal immunity that result in a disturbed intestinal balance [2]. An abnormal Th1 response is induced by dendritic cells that present commensal bacteria [3], which leads to overproduction of pro-inflammatory cytokines, including interferon-γ (IFN-γ) and tumor necrosis factor-alpha (TNF-α). In combination with impaired regulatory T cell (Treg) function, this is thought to lead to persistent inflammation in Crohn’s disease [4].

In about one third of patients, histopathology of biopsy specimens show granulomas; a feature supporting the diagnosis Crohn’s disease [5, 6]. As early as in the 1980s, a corona of B lymphocytes around the granuloma was described [7], which parallels granulomas in patients with sarcoidosis [8]. Furthermore, similar to patients with sarcoidosis [8–10], patients with Crohn’s disease show signs of abnormal B-cell responses that include increased numbers of immunoglobulin (Ig)-secreting cells [11], and serum antibodies against Saccharomyces cerevisiae antibodies (ASCA) and neutrophils (ANCA) [12, 13]. Being good antigen-presenters and cytokine producers, B cells can regulate T cell responses [14]. Indeed, B-cells were found to affect regulatory T cell through production of IL-10 [15]. However, it is not been clarified how B cells influence disease activity, because studies in murine models have reported ambiguous results, supporting either a suppressive or exacerbating role in gut inflammation [16–18].

In spite of a potential role of B cells in chronic inflammation, circulating naive B cells and class-switched memory B cells were found to be normally present in peripheral blood of patients with Crohn’s disease, whereas IgM memory B cell numbers were reduced [19]. IgM memory cells consist of two types; IgM-only (CD27^+^IgM^+^IgD^-^) and natural effector B cells (CD27^+^IgM^+^IgD^+^). While all IgM-only memory B cells originate from germinal center responses, about one-third of natural effector cells in healthy controls are derived from T-cell independent responses in the marginal zone of the spleen [20–22].

These contrasting observations did not clarify the exact role of B-cell involvement in Crohn’s disease. Therefore, we here aimed to elucidate their contribution in Crohn’s disease through detailed molecular analysis and immunophenotyping in locally inflamed intestinal tissue and in peripheral blood. Moreover, to evaluate candidate B-cell markers for monitoring therapeutic efficacy, we studied, the B-cell compartment after anti-TNFα therapy in patients treated with infliximab.

## Materials and Methods

### Patients

Clinical data and blood samples of 30 patients with Crohn’s disease and 28 healthy controls were collected after written informed consent was obtained (Table 1). In addition, surplus tissue materials from diagnostic colon biopsies of 5 patients were retrospectively analyzed. This study was performed according to the Declaration of Helsinki. This study was approved by the Medical Ethics Committees of Erasmus MC (ethics approval number MEC-2011-060) and Alfred Hospital (ethics approval number 472/15) and patients were recruited from the Ikazia Hospital in Rotterdam (The Netherlands) and the Alfred Hospital in Melbourne (VIC, Australia).

**Table 1:** Clinical and basic immunological characteristics of patients with Crohn’s disease.

Patient	Gender	Age (yr)	Disease duration (yr)	Medication	Surgery	Granuloma	B-cells	T-cells	NK-cells	IgG	IgG1	IgG2	IgG3	IgG4	IgA	IgA1	IgA2	IgM
1	F	30	0	None	No	Yes	**91**	1,200	270	ND	ND	ND	ND	ND	ND	ND	ND	ND
2	F	25	3	None	Yes	No	197	1,100	310	ND	ND	ND	ND	ND	ND	ND	ND	ND
3	M	23	4	None	No	No	208	1,575	220	ND	ND	ND	ND	ND	ND	ND	ND	ND
4	M	34	13	5-ASA	Yes	No	**430**	1,120	**430**	**6.8**	**4.8**	1.9	0.2	1.0	2.8	2.0	**1.4**	1.4
5	M	43	10	5-ASA	Yes	Yes	**470**	1,620	270	10.1	7.1	3.8	0.9	**<0.06**	1.7	1.2	0.2	0.7
6	M	63	5	5-ASA	Yes	No	160	**2,190**	310	10.0	6.2	4.8	0.8	0.4	**4.9**	**3.6**	**1.6**	0.7
7	M	35	22	None	Yes	No	**90**	**470**	220	9.0	5.2	5.3	0.6	0.1	**4.3**	**2.9**	**2.4**	0.7
8	M	33	2	5-ASA	No	Yes	220	1,260	190	11.1	8.3	2.7	0.9	0.5	3.6	**2.6**	**0.8**	0.5
9	F	22	7	None	No	No	250	1,110	270	14.9	11.1	4.0	0.8	0.4	2.6	1.8	0.3	2.3
10	F	27	0	5-ASA	No	Yes	**590**	1,790	230	9.2	5.7	3.7	0.5	1.2	1.8	1.3	0.3	1.1
11	F	62	21	5-ASA	Yes	Yes	160	1,090	220	7.8	**4.8**	3.7	0.5	**<0.06**	3.4	2.3	**1.0**	0.6
12	F	26	8	5-ASA	No	No	260	750	150	10.6	8.2	3.1	0.2	0.2	1.5	1.1	0.4	0.6
13	F	61	11	5-ASA	Yes	No	210	1,600	160	15.1	**13.0**	2.5	0.7	0.1	2.1	1.5	0.3	1.6
14	F	48	34	5-ASA	Yes	Yes	180	1,220	100	8.4	7.3	**0.9**	0.5	**<0.06**	2.7	1.8	**1.0**	0.7
15	M	45	6	5-ASA	No	No	380	**2,060**	**820**	**17.3**	10.7	**8.3**	0.4	**2.2**	**5.8**	**4.3**	**1.3**	0.7
16	F	35	1	None	No	No	333	**1,990**	250	10.7	7.1	5.3	0.6	**<0.06**	0.7	**0.5**	0.2	1.2
17	F	31	11	None	No	No	230	**3,250**	250	9.5	8.5	**1.4**	0.7	**<0.06**	1.3	1.0	0.4	1.3
18	F	35	8	None	No	Yes	350	1,120	180	**16.4**	**12.5**	6.0	1.0	0.2	3.3	2.4	0.5	1.4
19	F	41	23	5-ASA	Yes	No	110	1,330	150	8.8	5.8	4.1	0.4	**<0.06**	2.5	1.6	**1.1**	1.2
20	F	53	0	None	Yes	No	**870**	**2,500**	260	**6.3**	**4.3**	2.9	0.5	0.1	0.8	0.6	0.3	ND
21	M	39	3	5-ASA	No	No	**680**	1,580	**660**	15.8	**13**	3.5	0.8	1.1	1.6	1.1	0.4	1.0
22	F	34	14	IFX, 5-ASA	No	No	240	1,730	160	11.7	9.0	2.5	0.4	0.1	1.4	1.1	0.1	1.3
23	F	51	14	IFX, AZA	Yes	Yes	150	970	110	8.7	6.8	2.1	0.4	0.1	2.3	1.8	0.2	1.1
24	M	31	4	IFX	No	No	200	1,130	**80**	9.0	5.0	4.0	0.7	0.7	2.3	1.7	0.3	0.5
25	M	58	37	IFX, 5-ASA, AZA	Yes	No	200	**2,030**	**30**	**16.9**	**13.3**	2.8	0.6	0.1	2.6	1.9	0.2	0.9
26	F	48	19	IFX	Yes	No	370	**2,400**	**780**	9.6	5.1	4.4	0.4	**<0.06**	3.1	2.1	**1.4**	1.4
27	F	22	4	IFX	Yes	No	**790**	1,760	220	ND	ND	ND	ND	ND	ND	ND	ND	ND
28	F	33	15	IFX, AZA	Yes	Yes	128	**513**	**21**	ND	ND	ND	ND	ND	ND	ND	ND	ND
29	F	46	9	IFX	Yes	No	120	**460**	**28**	ND	ND	ND	ND	ND	ND	ND	ND	ND
30	M	22	2	IFX, AZA	No	No	199	855	**22**	ND	ND	ND	ND	ND	ND	ND	ND	ND

Lymphocyte subsets are provided in cells/μL, Immunoglobulin levels in g/L. Abnormal values are depicted in bold font. Definition of abbreviations: F, female; M, male 5-ASA, 5-aminosalicylic acid; IFX, infliximab; AZA, azathioprine. Normal values: B cells: 100–400 cells/μl; T cells: 700–1900 cells/μl; NK cells; 100–400 cells/μl; IgG: 7–16 g/L; IgG1: 4.9–11.4 g/L; IgG2: 1.50–6.4 g/L; IgG3: 0.20–1.10 g/L; IgG4: 0.080–1.40 g/L; IgA: 0.70–4.0 g/L; IgA1: 0.6–2.4 g/L; IgA2: 0.1–0.6 g/L; IgM 0.4–2.3 g/L.

### Immunohistochemistry of gut tissue biopsies

Tissue slides were stained with hematoxylin and eosin. Immunohistochemistry was performed using monoclonal antibodies against CD4 (clone SP35), CD3 (2GV6), CD79a (SP18; all from Ventana, Tucson, AZ), CD8 (C8/144 B), CD20 (L26), IgG (rabbit polyclonal; all from Dako Cytomation, Glostrup, Denmark), IgA (rabbit polyclonal, Cell Marque, Rocklin, CA), CD138 (B-A38; IQ Products, Groningen, The Netherlands), and IgM (IgM88; Biogenex, Fremont, CA).

### Flowcytometry and cell sorting of blood lymphocytes

Absolute counts of blood CD4 and CD8 T cells, CD16^+^/56^+^ natural killer cells, and CD19^+^ B cells were obtained with a diagnostic lyse-no-wash protocol. Eight-color flow cytometric analysis was performed as described previously to detect transitional, naive mature, six memory B cell subsets, plasmablasts and CD21^low^CD38^dim^ cells (S1 Fig) on a 3-laser FACS LSRII with standardized configuration according to Euroflow protocols (BD Biosciences, San Jose, CA) [23]. Detailed analysis of B cell subsets was performed with IgM-HorV450 (G20-127; BD), IgD-biotin (IA6-2), IgG-PE (G18-145), CD19-PE-Cy7 (SJ25C1), CD19-PerCP-Cy5.5 (SJ25C1), CD21-PE-Cy7 (B-ly4), CD27-PerCP-Cy5.5 (L128), CD27-APC (L128), CD38-APC-H7 (HB7; all from BD Biosciences, San Jose, CA, USA) and IgA-FITC (IS11-8E10; Miltenyi-Biotec GmbH, Germany) [24]. Biotinylated antibodies were visualized with streptavidin-Pac.Orange (Invitrogen).

Naive mature and natural effector B cells were high-speed cell sorted to greater than 95% purity on a FACSAria I (BD Biosciences), as described previously [25].

### Quantification of serum immunoglobulin levels

Serum IgM, IgG, and IgA levels were measured with an immunoturbidimetric method (Hitachi Analyzer; Roche, Basel, Switzerland). IgG and IgA subclasses were determined using the immunonephelometric method (Sanquin, Amsterdam, The Netherlands).

### Molecular analysis of replication history and immunoglobulin heavy chain (*IGH*) transcripts

*IGHA* and *IGHG* transcripts were amplified from PBMC cDNA of patients with Crohn’s disease (n = 4) and healthy controls (n = 4). *IGHV3* and *IGHV4* leader primers and consensus Cα or Cγ reverse primers were used [22].

DNA was isolated from sorted naive mature and natural effector B cells of patients with Crohn’s disease (n = 4) to analyze the replication history with the kappa-deleting recombination excision circle assay as described previously [25]. In addition, *IGH* gene rearrangements were amplified from DNA of sorted natural effector B cells. PCR products were cloned into the pGEM-T easy vector (Promega, Madison, WI) and prepared for sequencing on an ABIPRISM 3130XL (Applied Biosystems, Carlsbad, CA). Obtained sequences were analyzed with IMGT database (http://imgt.cines.fr), Joinsolver (https://joinsolver.niaid.nih.gov) and Bayesian estimation of Antigen-driven SELectIoN (BASELINe; http://selection.med.yale.edu/baseline/). IgA and IgG receptor subclasses were determined using the *IGH* reference sequence (NG_001019).

### Statistics

Statistical analyses were performed using the Mann-Whitney test (SPSS version 18.0), χ2 test or Spearman correlation as indicated in Figure legends. A P-value <0.05 was considered statistically significant.

## Results

### Clinical and basic immunological characterization of patients

In this study, 30 patients with biopsy-confirmed Crohn’s disease (11 males) were included with a mean age of 38.5 yr (range 22–62 yr; Table 1). Patients 1–21 had not received immunosuppressive drugs for at least three months prior to inclusion. Of these 21 patients, 12 patients received 5-ASA medication, 9 were without any medication for Crohn’s disease and 11/21 patients had received systemic immunosuppressive medication in the past. All patients had clinically mild to moderate disease without a need for systemic immune suppressive treatment at the time of study inclusion. Patients 22–30 received infliximab treatment for >6 weeks at study inclusion and were clinically good responders. In 9/30 patients, granulomas were previously detected in ileal or colon biopsies and 16/30 patients had a history of surgical resection of the gut. The average duration of disease at study inclusion was 10.3 years (range 0–34 year). Mean values of B, T and NK cells, as well as mean serum IgM, IgG and IgA levels were within the normal range. The average IgA2 serum level of the patients was increased as compared to controls (0.7 g/L; range 0.1–2.4; normal range 0.1–0.6), with 9/23 patients having levels above the normal range.

### B cell localization around granulomas

**Fig 1:**
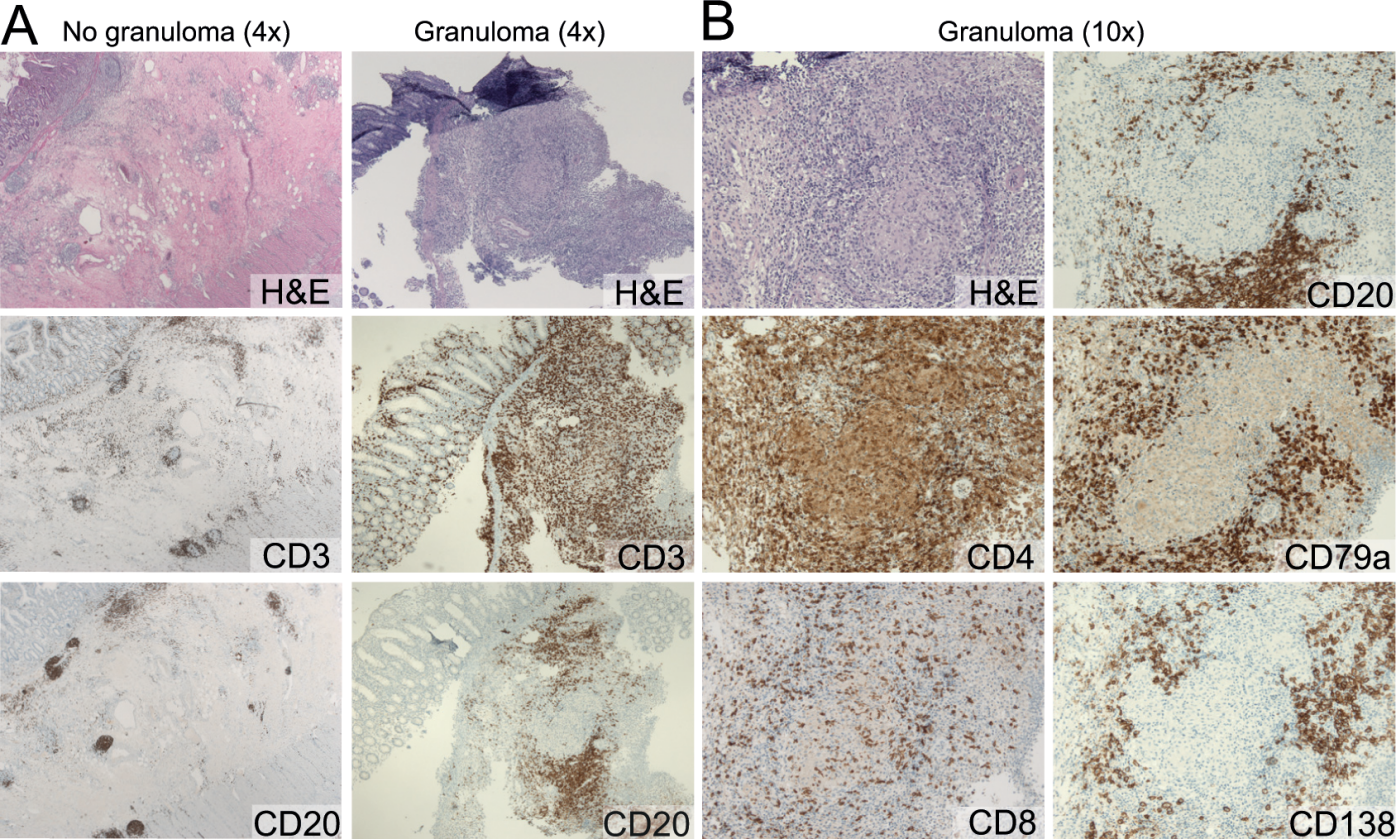
B-cells accumulate around granulomas in affected colon tissue in Crohn’s disease. **A,** Representative images of colon biopsies with granulomas and non-granulomatous tissue in two patients with Crohn’s disease. **B,** Magnifications of granulomatous tissue from panel A.

All colon tissue biopsies showed inflammation compatible with Crohn’s disease. Haematoxylin and eosin-staining of the granulomas did not show any sign of necrosis. T cells were easily detectable with stainings for CD3, CD4 or CD8, and were located throughout the inflamed tissue, both inside and outside the granulomas (Fig 1). In agreement with previous observations [7], CD4^+^ T cells were more numerous than CD8^+^ T cells with a ratio of 4:1. CD20^+^ B cells were detectable in intestinal biopsies, but these were restricted to normal lymphoid follicles and were very sparse in the non-granulomatous inflamed tissue (Fig 1). However, directly surrounding the granulomas, B cells were numerous as visualized with CD20 or CD79a stainings (Fig 1). Plasma cells are abundantly present in the human gut tissue of healthy individuals, with IgA as their major product [26, 27]. Tissue sections from our patients with Crohn’s disease showed numerous CD138^+^ plasma cells, both in inflamed and in non-inflamed regions. These plasma cells were not specifically localized near granulomas, but were distributed over the gut tissue with the majority secreting IgA and smaller fractions IgG or IgM (S2 Fig). The specific localization of B cells surrounding granulomas indicates involvement of B cells in the immunopathogenesis of granulomatous inflammation in Crohn’s disease.

### Abnormalities in blood B cell subsets in patients with Crohn’s disease

**Fig 2:**
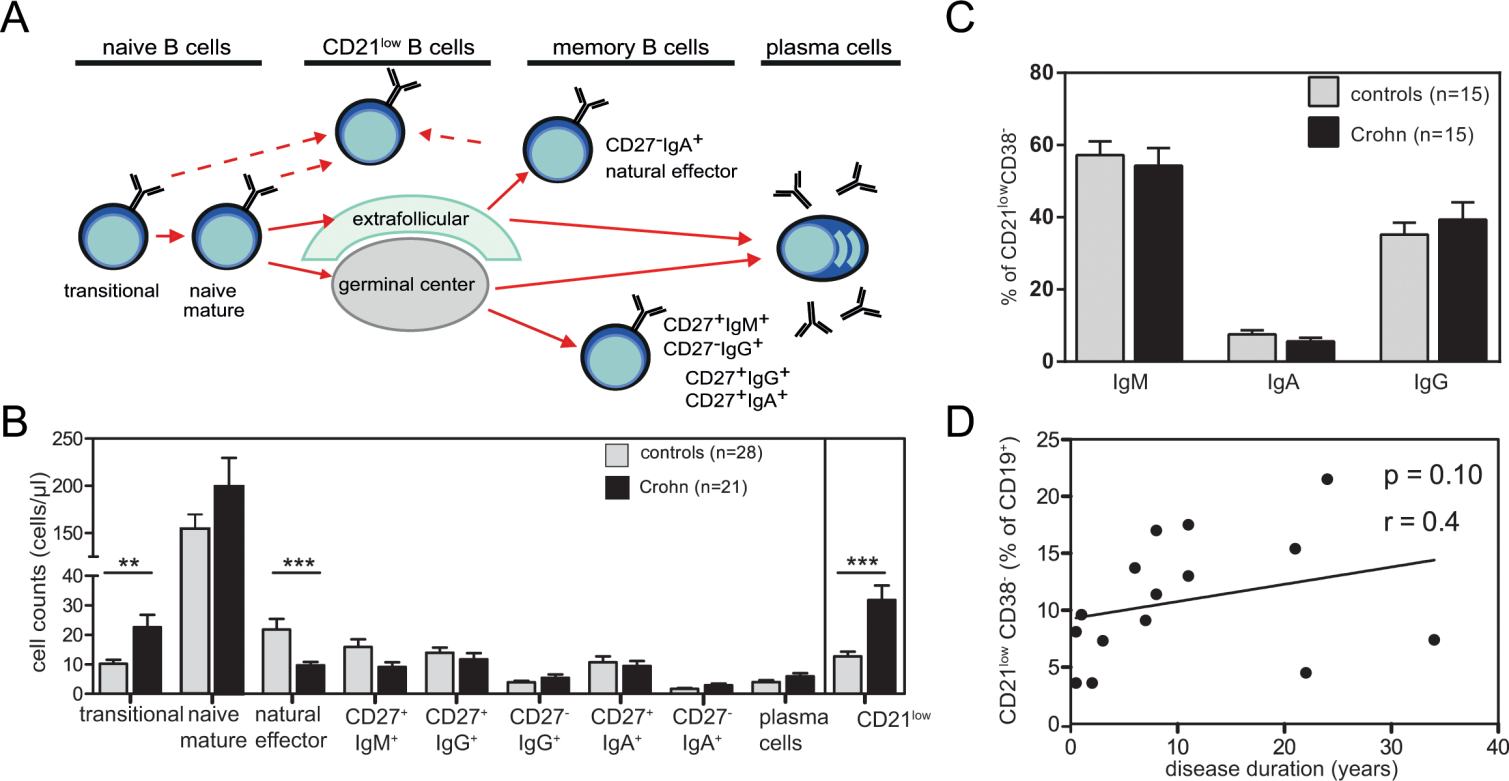
Composition of the blood B-cell compartment in patients with Crohn’s disease. **A.** Schematic overview of peripheral B-cell subsets. **B.** Average numbers of blood B cell subsets of 21 patients affected with Crohn’s disease (black bars) and 28 healthy controls (light grey bars). **C.** Distribution of IgM, IgA and IgG within CD21^low^ in patients and controls **D.** Total CD21^low^ B cells in relation to disease duration. Statistical analyses were performed with the Mann-Whitney test or Spearman correlation; *, P<0.05; **, P<0.01.

To study whether local intestinal inflammation affected B cells systemically, we studied blood B-cell subsets in 21 patients with Crohn’s disease. Flowcytometric analysis revealed normal numbers of total CD19^+^ B-cells in patients (n = 21) as compared with healthy controls (n = 28). Further subsetting of these CD19^+^ B cells (Fig 2A) revealed significantly increased numbers of CD38^high^CD24^high^ transitional B cells (P = 0.009), while CD27^-^IgD^+^ naive mature B cells were normally present (Fig 2B). Within the antigen-experienced B-cell compartment, IgM^+^ memory B cells were low with CD27^+^IgM^+^IgD^+^ natural effector B cells being significantly decreased (P<0.001), IgM-only B cells (CD27^+^IgM^+^IgD^-^) not-significantly decreased (P = 0.06). The numbers of class-switched B cell subsets (CD27^+^IgG^+^, CD27^-^IgG^+^, CD27^+^IgA^+^ and CD27^-^IgA^+^) and plasma blasts were similar between patients and controls. Large fractions of the patients’ B cells showed low CD21 expression levels and these numbers were significantly higher than in healthy controls (P<0.0001). The increase was not related to disease duration (P = 0.10; Fig 2D), and a large fraction of these CD21^low^ B cells were Ig class switched to IgA or IgG, suggestive of an origin from antigen-experienced B cells (Fig 2C).

To study whether the abnormalities in transitional, natural effector and CD21^low^ B cells were associated with surgical treatment and current or past medication, additional analyses were performed following division of the total 21 patients into patients with (n = 10) or without surgical resection (n = 11), into patients currently treated with (n = 12) or without 5-ASA medication (n = 9) and patients with (n = 11) and without (n = 10) a history of systemic medication. All three analyses revealed similar patterns for the separate patient groups (S3 Fig), thereby excluding differential effects of these treatments on the blood B-cell compartment.

### Impaired generation of natural effector B cells

**Fig 3:**
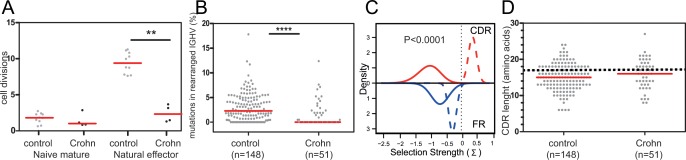
Replication history and SHM levels in *IGHV* genes of natural effector B cells. **A,** Replication history of naïve and natural effector B cells as assessed using the KREC assay [25]. **B,** *IGHV* mutation frequencies in rearranged *IGH* genes of natural effector B cells in patients and controls (total numbers of sequences indicated between brackets). Grey dots represent unique sequences; red lines represent median values. **C**, Selection for replacement mutation in IGHV-CDR (red line) and IGHV-FR regions (blue lines) as determined with the BASELINe program [28, 29]. Solid lines represent patients; dashed lines represent healthy controls. Selection Strengths >0 indicate positive selection. **D,** IGH-CDR3 size distributions. All individual sizes are indicated as grey dots, red lines representing median values. The dashed line represents median values for centroblasts and centrocytes. Sorted cells were analyzed from patients 15, 16, 18 and 19. Controls were published previously [30, 31]. Statistical analysis was performed with the Mann-Whitney test; *, P<0.05; **, P<0.01; ***, P<0.001; ****, P<0.0001.

Our flowcytometric analysis showed decreased numbers of natural effector B cells in peripheral blood of patients with Crohn’s disease, line with previous findings [19]. To study whether the decline was due to impaired generation of these cells, we analyzed the replication history and somatic hypermutation (SHM) levels in purified cells from four patients (Patient 15, 16, 18 and 19). Naive mature B cells of patients and controls showed a similar replication history of 1–2 cell divisions [25]. However, the patients’ natural effector B cells showed a replication history of only 2 cell division versus 9 in controls (P = 0.002; Fig 3A). These natural effector B cells carried diverse *IGH* gene rearrangements, with shorter *IGH*-CDR3 sizes than in naive B cells, which is a typical feature of antigen-experienced B cells (Fig 3D) [22]. Still, the majority of rearrangements amplified from the patients carried unmutated *IGHV* genes (28 of 51 unique rearranged *IGHV*). Moreover, the overall SHM levels were significantly lower than in controls (P<0.0001; Fig 3B), and hardly higher than in naive mature B cells. The few mutations in patients’ Ig genes were normally targeted (S1 Table). However, on top of their low numbers, the mutations in complementarity determining regions (CDR) were not selected for amino acid replacements as is typical seen in healthy controls (P<0.0001; Fig 3C). Thus, IgM^+^IgD^+^-expressing memory B cells in patients with Crohn’s disease are not only decreased in number, they also display a defects in replication history and SHM.

### Increased SHM levels in Ig genes of switched memory B cells

In contrast to IgM^+^ memory B cells, Ig class-switched memory B cells were normally present in blood of patients with Crohn’s disease (Fig 2) [19]. Their IgA and IgG transcripts displayed a diverse usage of *IGHV3* and *IGHV4* subgroups with CDR3 size distributions similar to those of controls and typical for antigen-experienced B cells with a median of 15 amino acids (S5 Fig). These transcripts showed high levels of SHM, which appeared to be normally targeted to the typical sequence motifs (S1 Table). In addition, nucleotide substitution spectra and transition/transversion ratios did not differ between patients and controls. To determine whether these transcripts showed signs of antigen selection, we analyzed selection for replacement mutations using the BASELINE program. Similar to healthy controls, sequences derived from patients with Crohn’s disease showed positive selection for replacement mutations in CDR and negative selection in framework regions (FR) (S5C Fig).

**Fig 4:**
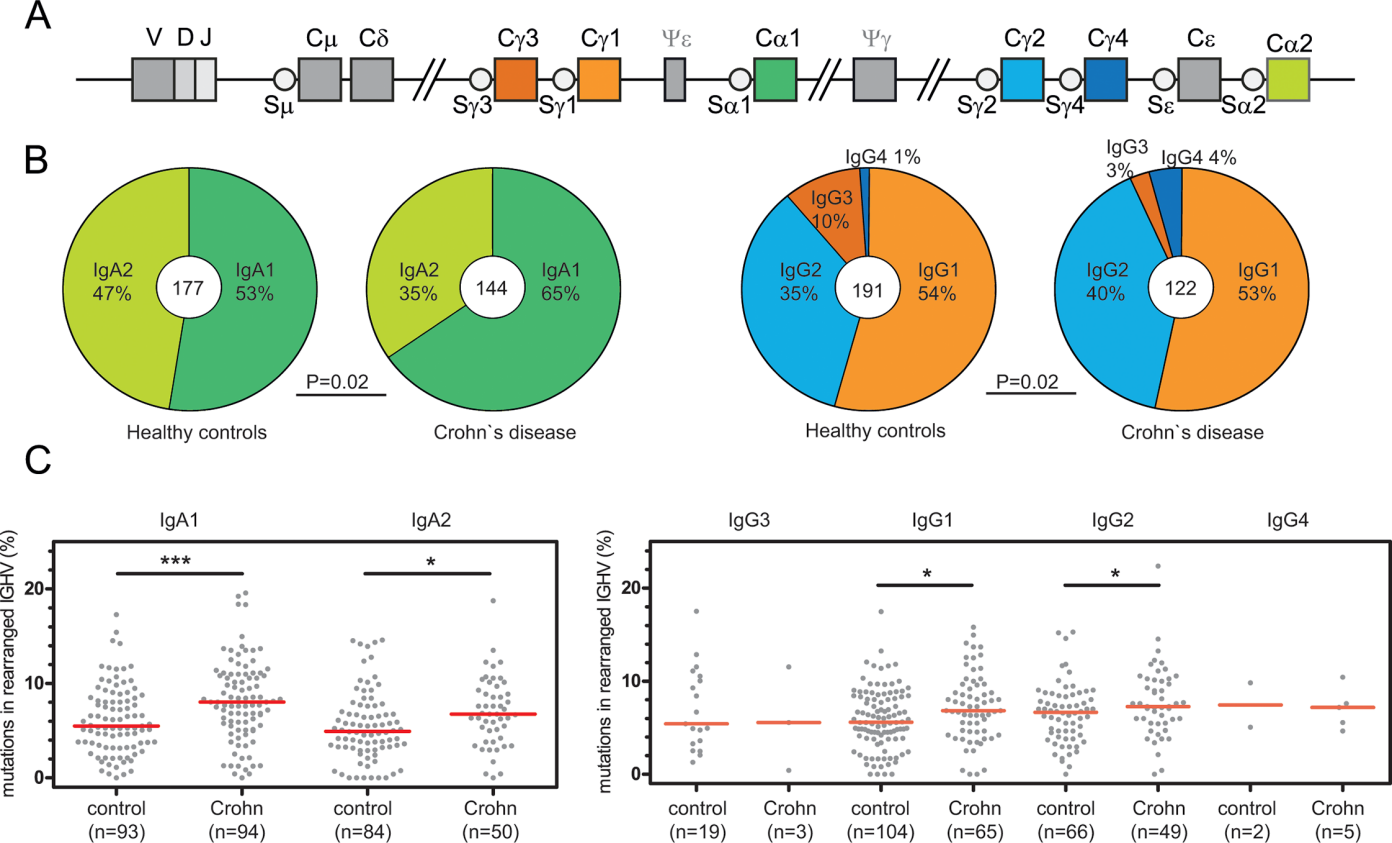
IgA and IgG subclass analysis. **A,** Schematic representation of the constant region of the human IGH locus. **B,** Distribution of IgA and IgG subclass use in switched transcripts of healthy controls and patients with Crohn’s disease. Total numbers of analyzed sequences are indicated in the middle of the plots. χ^2^ Test was performed to analyze differences in distributions. **C,** Combined *IGHV* mutation frequencies in IgA and IgG transcripts in patients and controls (total numbers of sequences indicated between brackets). Grey dots represent unique sequences; red lines represent median values. Statistical analysis was performed with the Mann-Whitney test; *, P<0.05; **, P<0.01; ***, P<0.001.

To study whether the high SHM levels were the result of altered IgG and IgA subclass usage, we analyzed these in the rearranged transcripts [32, 33]. Patients with Crohn’s disease showed increased IgA1 and IgG2 usage, to the expense of IgA2 and IgG3 (Fig 4B). Still, these altered distributions did not underlie the difference in SHM levels. IgA1 and IgA2, as well as IgG1 and IgG2 transcripts of the patients carried more SHM than those of controls (Fig 4C). More specifically, a substantial fraction of IgA2 transcripts from controls was hardly mutated, and this fraction was nearly absent in patients with Crohn’s disease. In conclusion, patients with Crohn’s disease show increased levels of SHM with otherwise normal targeting and selection for replacement mutations. This was independent of the concomitant reduction in IgA2 and IgG3 subclass usage.

### Normalization of blood B-cell subsets in infliximab-treated patients

**Fig 5:**
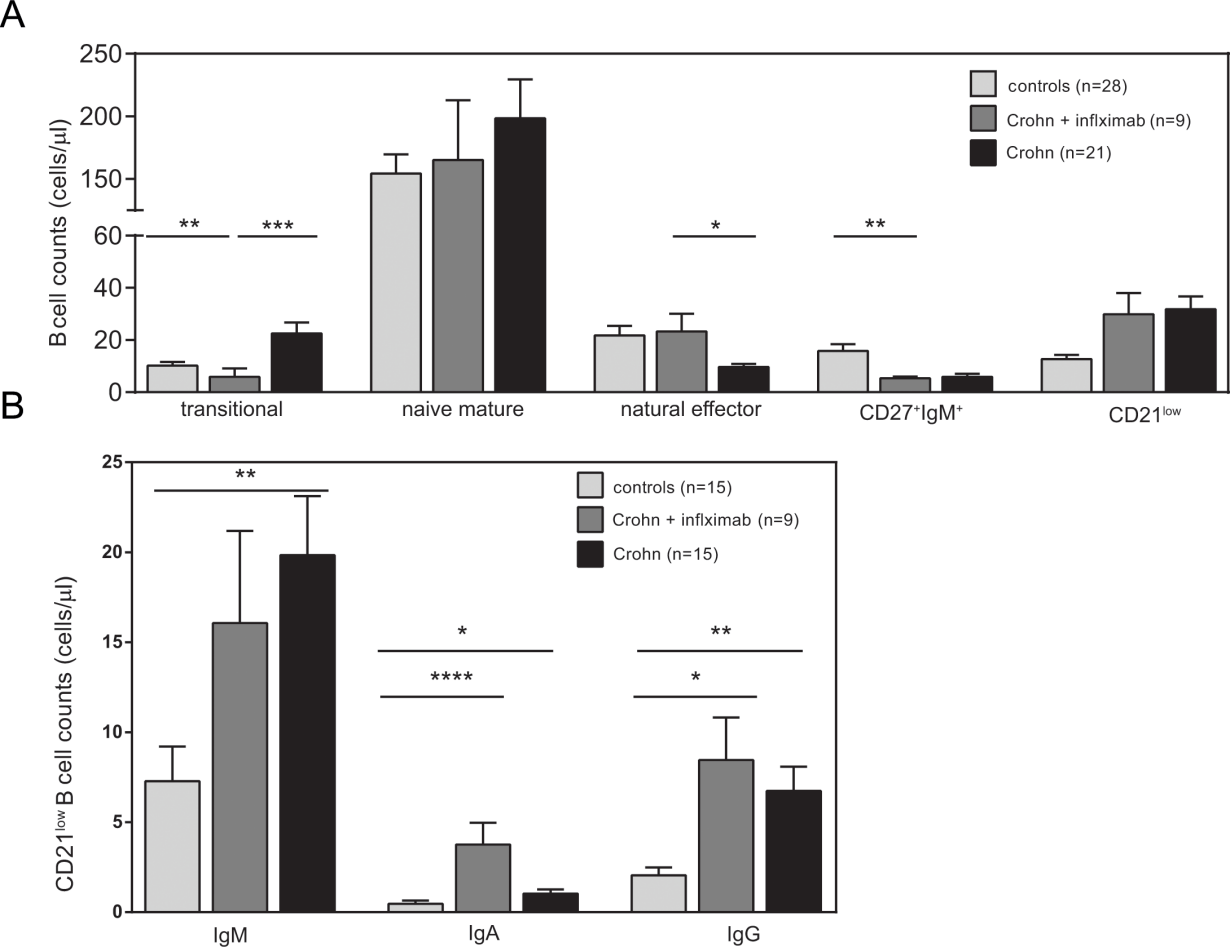
Effects of infliximab on blood B-cell and CD21^low^ compartment. **A**, Blood B-cell compartments in patients under treatment with infliximab. **B,** Absolute total numbers of IgM, IgA and IgG with low CD21 expression in controls, patients and patients under treatment with infliximab. Bars represent mean values ±SEM. Statistical analysis was performed with the Mann-Whitney test; *, P<0.05; **, P<0.01; ***, P<0.001.

Previous observations indicated normalization of spleen function and levels of circulating IgM+ memory B cells in Crohn’s disease patients following infliximab therapy [34]. To study whether successful treatment normalized the total peripheral B-cell compartment, we phenotyped blood B cells in 9 patients that were receiving infliximab (patients 21–30; Table 1). Infliximab was administered once every eight weeks for a long period of time (range 8 months-10 years), and all patients were in clinical response after treatment. In contrast to patients not receiving infliximab, transitional B cells and natural effector B cells were normalized to levels comparable with healthy controls (Fig 5A). However, IgM-only B cell numbers were still low, and CD21^low^ B cells remained increased as compared to healthy controls. Within CD21^low^, the IgM, IgA and the IgG expressing subsets were higher in number than in healthy controls. Patients treated with infliximab showed a further increase in the IgA subset, with an accompanied (non-significant) decrease in IgM (Fig 5B). A substantial fraction of the CD21^low^ subset expressed CD27, and CD27^+^ and CD27^-^ was similar between controls and patients (S4A Fig). In absolute numbers, both fractions were elevated in patients. Thus, patients with Crohn’s disease show systemic abnormalities in their B cell compartments, which appear almost completely recovered by successful infliximab treatment.

## Discussion

In this study, we demonstrate that patients with Crohn’s disease have an infiltration of B cells around granulomas and an altered B-cell compartment in the peripheral blood. While IgM memory B-cell formation was impaired, Ig class switched B cells showed molecular sings of chronic stimulation. Importantly, alterations in the peripheral B-cell compartment normalized after treating inflammation effectively with TNFα-blockers.

Our findings of B cells surrounding granulomas in Crohn’s disease extend previous observations from the 1980s [7], and more recent ones from pediatric patients with Crohn’s disease with *NOD2* gene mutations [35]. These B cells appear to be lymphocytes and not plasma cells and localize specifically around the granulomas. Still, the origin and types of B cell subsets remain unclear. Large amounts of B cells were also found to be present around the granulomas in sarcoidosis [8], and consequently researchers have evaluated treatment with anti-CD20 therapy (e.g. with rituximab) [36]. It is possible that these B cells are crucial for the formation of granulomas. This is supported by studies in mouse models that were capable of granuloma formation in the absence of T cells, but not in the absence of B cells [37]. Furthermore, granulomas are found in a large fraction of patients with antibody deficiencies in the presence of B cells (esp. Common Variable Immunodeficiency; CVID), while these have not been reported in patients with X-linked agammaglobulinemia, who lack circulating B cells due to a block in differentiation of precursor B cells [38]. How these B cells would function in the formation of granulomas remains unclear. B-cell depletion therapy seems to induce and exacerbate colitis [39, 40], while immunoglobulin substitution can induce rapid dampening of inflammation in patients with Crohn’s disease [41]. Thus, it is well-possible that the local B cells have a regulatory function to control inflammation [42].

Our patients showed alterations in blood B cell subsets in absence of systemic immunosuppressive therapy. One of these was a strong reduction in circulating IgM+ memory B cells, which was the result of impaired generation rather than increased loss, because the few remaining IgM memory ‘natural effector’ B cells showed severely reduced replication history, SHM levels and absence of selection for replacement mutations in CDR. The loss of IgM memory B cells was previously attributed to impaired spleen function [19]. However, a large fraction of these ‘natural effector’ B cells is dependent on T-cell help and more likely originates from germinal center reactions [21, 22]. Considering the strongly decreased natural effector B-cell numbers in our patients, it is therefore likely that in addition to IgM responses in the spleen, also germinal center responses are impaired in the generation of IgM^+^ memory B cells in patients with Crohn’s disease.

In contrast to IgM^+^ memory, transitional and CD21^low^ B-cell numbers were increased in our patients. Higher numbers of transitional cells were previously observed in patients with other chronic inflammatory diseases, including sarcoidosis and SLE [8, 9, 43]. This increase could reflect increased B-cell output from the bone marrow. Still, this did not result in higher numbers of circulating mature B cells and might be due to inability of these transitional B cells to further mature. CD21^low^ B cells are peculiar cells that have been described to be functionally anergic with the downregulation of CD21 suppressing their responsiveness and decreasing their survival [22, 44]. The increase in transitional B cells could therefore be a compensation for the loss of mature B cells through downregulation of CD21. While CD21^low^ B cells are scarce in healthy controls, their numbers are increased during infections, autoimmune diseases [22, 45, 46], CVID with autoimmunity and Down syndrome [44, 46–48]. As these cells were not increased in patients with sarcoidosis [8], CD21^low^ B cells could represent a marker of distinct pathophysiology between these two granulomatous inflammatory diseases.

The numbers of natural effector B cells normalized under infliximab therapy, an observation that was made previously as well and was associated with restoration of spleen function [34]. More recently, Li and colleagues also confirmed these low numbers of pre-switched memory B cells in inflammatory bowel disease and its restoration with TNFα-blockers [49]. Thus, infliximab therapy either directly or indirectly by dampening inflammation restores IgM memory in patients with Crohn’s disease. Whether natural effector B cells can predict successful therapeutic outcome would need to be investigated in future studies with longitudinal follow-up of patients. Treatment with 5-ASA did not show this effect on the B-cell compartment. This could be due to the difference in therapeutic mechanisms or the merely local application of 5-ASA medication in contrast to systemic effects of infliximab. Alternatively, infliximab can induce and maintain mucosal healing [50]. Furthermore, the CD21^low^ population was the aberrant B-cell subset in our patient group that did not normalize during treatment with infliximab, suggesting that the process to downregulate CD21 is either not affected by TNFα-blockers, or is maintained to dampen inflammation.

A large fraction of CD21^low^ B cells was Ig class switched, suggesting their origin from memory B cells. Indeed, the increased SHM levels in IgA and IgG transcripts reflected abnormally high or strong activation of these class-switched memory cells. SHM levels are tightly regulated and even in individuals continuously exposed to parasites these are not increased [32, 33]. Notably, IgA transcripts in patients with Crohn’s disease were highly mutated, and the frequencies of hypomutated transcripts were lower than in healthy controls. A substantial fraction of blood IgA+ memory B cells carries polyreactive immunoglobulins. These are typically highly mutated and bind strongly to mucosa-colonizing bacteria [51]. Despite the high SHM levels, the Ig transcripts from patients with Crohn’s disease did not show signs of additional selection for replacement mutations in CDR regions. This is suggestive of a lack in additional affinity maturation, and the result of abnormal and chronic activation in patients with Crohn’s disease and in previously studied sarcoidosis patients [8]. Despite the signs of chronic stimulation, total numbers of IgA and IgG memory B cells were not increased in blood of patients with Crohn’s disease. This is potentially due to their infiltration into tissue. Alternatively, these cells could be silenced by downregulating CD21 expression. This would make the cells more susceptible to cell death and would explain, at least in part, the expansion of the CD21^low^ B cell population.

## Conclusion

Our study demonstrates distinct B-cell maturation alterations in both local inflamed tissue and in peripheral blood of patients with Crohn’s disease. These effects were independent of 5-ASA treatment or past systemic therapy and surgical resections, and seemed homogeneous in our study population. Especially the Ig class-switched B cells show signs of chronic stimulation, while the generation of IgM memory B cells is impaired. Moreover, clinical improvement is heralded by normalization of the elevated circulating transitional and natural effector B cells in response to TNFα-blockers. Thus, through dissection of the local and systemic B cell compartments, this study provides new insights into their role in chronic inflammation. Specifically, blood B-cell deviations could represent good markers to predict treatment success before or early after start of infliximab or other novel therapeutics in Crohn’s disease.

## Supporting Information

S1 Fig **Flowcytometric gating strategy for B-cell subsets in a representative healthy control (A) and a patient with Crohn’s disease (B)**. B cells defined as CD19+ lymphocytes and further subsetted into two naive subsets (transitional cells and naive mature cells), six memory subsets, plasma cells and CD38^dim^C21^low^ cells. Non-switched memory B cells were separated into natural effector (CD27^+^IgM^+^IgD^+^) and IgM-only cells (CD27^+^IgM^+^IgD^-^). IgA and IgG switched memory B cells were further separated into CD27^-^ and CD27^+^ subsets.
(EPS)

S2 Fig **Immunohistological analysis of plasma cells in sections with (A) and without granulomas (B) in colon biopsies of two patients with Crohn’s disease.** Both tissues show presence of CD138^+^ plasma cells, with the majority producing IgA, to a lesser extent IgG and low frequencies IgM.
(TIF)

S3 Fig **Blood B-cell compartments in (A) patients with a history of resection, (B) patients under treatment with 5-ASA medication, and (C) patients with a history of systemic immunosuppressive medication.** Bars represent mean values ±SEM. No significant differences were found for any subset between Crohn’s patients with or with the indicated mode of treatment (Mann-Whitney U test).
(EPS)

S4 Fig CD21^low^ population in controls, patients on infliximab and Crohn’s disease patients without systemic treatment. **A,** Relative distribution of CD27^-^ and CD27^+^ cells within the CD21^low^ compartment. **B,** Absolute cell counts of CD27^-^ and CD27^+^ cells within CD21^low^ compartment. Bars represent mean values ±SEM.
(EPS)

S5 Fig Somatic hypermutation analysis of IgM, IgA and IgG B cells. **Somatic hypermutation levels in IGHV genes of rearranged IgA (A) and IgG (B) transcripts of four patients with Crohn’s disease and four healthy controls.** Grey dots represent unique sequences; red lines represent median values. **C,** Selection for replacement mutation in IGHV-CDR (red line) and IGHV-FR regions (blue lines) as determined with the BASELINe program [28, 29]. Solid lines represent patients; dashed lines represent healthy controls. Selection Strengths >0 indicate positive selection. **D**, IGH-CDR3 size distributions. All individual sizes are indicated as grey dots, red lines representing median values. The dashed line represents median values for centroblasts and centrocytes from controls (22).
(EPS)

S1 Table Targeting and selection of individual mutations in rearranged IGHV. (DOCX)
