# Supplementary figures and images for: B-Cell Dysregulation in Crohn's Disease Is Partially Restored with Infliximab Therapy
Source: PLoS One. 2016 Jul 28;11(7):e0160103. doi: 10.1371/journal.pone.0160103 (PMC4965034; doi:10.1371/journal.pone.0160103)

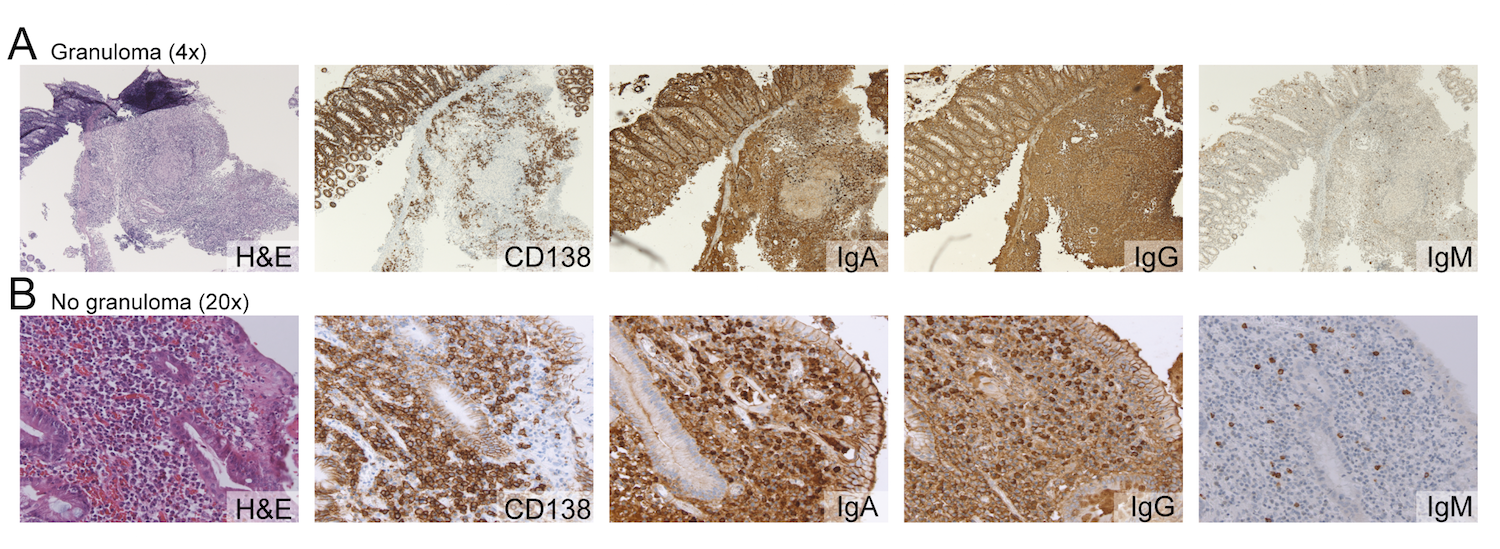

Supplement: S2 Fig — Immunohistological analysis of plasma cells in sections with (A) and without granulomas (B) in colon biopsies of two patients with Crohn’s disease. Both tissues show presence of CD138+ plasma cells, with the majority producing IgA, to a lesser extent IgG and low frequencies IgM. (TIF) [file pone.0160103.s002.tif]
