# Supplementary material for: B-Cell Dysregulation in Crohn's Disease Is Partially Restored with Infliximab Therapy
Source: PLoS One. 2016 Jul 28;11(7):e0160103. doi: 10.1371/journal.pone.0160103 (PMC4965034; doi:10.1371/journal.pone.0160103)
Supplement: S1 Table — (DOCX) [file pone.0160103.s006.docx]

|  | Natural effector | | | | IgA Memory | | | | IgG Memory | | | |
| --- | --- | --- | --- | --- | --- | --- | --- | --- | --- | --- | --- | --- |
|  | Control (n=120) | | Crohn (n=51) | | Control (n=112) | | Crohn (n=144) | | Control (n=129) | | Crohn (n=123) | |
| Mutated rear. (%) | 100/120 | (83.3) | 39/51 | (76.5) | 111/112 | (99.1) | 142/144 | (98.6) | 126/129 | (97.7) | 121/123 | (98.4) |
| Transitions (%) | 504/909 | (55.4) | 123/224 | (54.9) | 997/1850 | (53.9) | 1779/3503 | (50.8) | 1241/2438 | (50.9) | 1313/2653 | (49.5) |
| Transversions (%) | 405/909 | (44.6) | 101/224 | (45.1) | 853/1850 | (46.1) | 1724/3503 | (49.2) | 1197/2438 | (49.1) | 1340/2653 | (50.5) |
| Transitions at C·G (%) | 298/543 | (54.9) | 64/129 | (49.6) | 563/1076 | (52.3) | 991/2000 | (49.6) | 743/1432 | (51.9) | 777/1552 | (50.1) |
| Targeting of C·G (%) | 543/909 | (59.7) | 129/224 | (57.6) | 1076/1850 | (57.7) | 2000/3503 | (57.1) | 1432/2438 | (58.7) | 1552/2653 | (58.3) |
| RGYW (%) | 244.1/909 | (26.9) | 57.1/224 | (25.5) | 483.3/1850 | (26.1) | **774.6/3503** | **(22.1)*** | 613.7/2438 | (25.2) | **584.8/2653** | **(22.0)*** |
| WRCY (%) | 132/909 | (14.5) | 28.3/224 | (12.6) | 264.6/1850 | (14.3) | 536.8/3503 | (15.3) | 351.7/2438 | (14.4) | 372.5/2653 | (14.0) |
| WA (%) | 131.7/909 | (14.5) | 29.5/224 | (13.1) | 252.3/1850 | (13.6) | 449.6/3503 | (12.8) | 303.7/2438 | (12.5) | **164.3/2653** | **(13.7)*** |
| TW (%) | 45.2/909 | (5.0) | 15.4/224 | (6.9) | 151.9/1850 | (8.2) | 243/3503 | (6.9) | 159.0/2438 | (6.5) | 171.3/2653 | (6.5) |
| FR (R/S) | 379/212 | (1.8) | **69/63** | **(1.5)*** | 719/460 | (1.6) | 1519/954 | (1.6) | 1065/618 | (1.7) | 1150/697 | (1.6) |
| CDR (R/S) | 259/59 | (4.4) | 51/14 | (3.6) | 535/134 | (4.0) | 780/238 | (3.3) | 596/159 | (3.7) | 645/158 | (4.1) |
| FR denotes framework region; CDR, complementarity determining region; R/S is the ratio between replacement (R) and silent mutations (S); the numbers of analyzed sequences are indicated in brackets next to the population name. All analyses were performed with the JOINSOLVER™ program and the differences between controls and patients were analyzed with the χ2 test. *, P<0.05 | | | | | | | | | | | | |

**S1 Table. Targeting and selection of individual mutations in rearranged *IGHV***
